# Thickness-Dependent Gilbert Damping and Soft Magnetism in Metal/Co-Fe-B/Metal Sandwich Structure

**DOI:** 10.3390/nano14070596

**Published:** 2024-03-28

**Authors:** Yimo Fan, Jiawei Wang, Aitian Chen, Kai Yu, Mingmin Zhu, Yunxin Han, Sen Zhang, Xianqing Lin, Haomiao Zhou, Xixiang Zhang, Qiang Lin

**Affiliations:** 1 College of Science, Zhejiang University of Technology, Hangzhou 310023, China; 2 Key Laboratory of Electromagnetic Wave Information Technology and Metrology of Zhejiang Province, College of Information Engineering, China Jiliang University, Hangzhou 310018, China; 3 Physical Science and Engineering Division, King Abdullah University of Science and Technology, Thuwal 23955-6900, Saudi Arabia; 4 College of Science, National University of Defense Technology, Changsha 410073, China

**Keywords:** Co-Fe-B alloy film, spin dynamic, Gilbert damping, co-planar waveguide FMR, spintronics

## Abstract

The achievement of the low Gilbert damping parameter in spin dynamic modulation is attractive for spintronic devices with low energy consumption and high speed. Metallic ferromagnetic alloy Co-Fe-B is a possible candidate due to its high compatibility with spintronic technologies. Here, we report thickness-dependent damping and soft magnetism in Co-Fe-B films sandwiched between two non-magnetic layers with Co-Fe-B films up to 50 nm thick. A non-monotonic variation of Co-Fe-B film damping with thickness is observed, which is in contrast to previously reported monotonic trends. The minimum damping and the corresponding Co-Fe-B thickness vary significantly among the different non-magnetic layer series, indicating that the structure selection significantly alters the relative contributions of various damping mechanisms. Thus, we developed a quantitative method to distinguish intrinsic from extrinsic damping via ferromagnetic resonance measurements of thickness-dependent damping rather than the traditional numerical calculation method. By separating extrinsic and intrinsic damping, each mechanism affecting the total damping of Co-Fe-B films in sandwich structures is analyzed in detail. Our findings have revealed that the thickness-dependent damping measurement is an effective tool for quantitatively investigating different damping mechanisms. This investigation provides an understanding of underlying mechanisms and opens up avenues for achieving low damping in Co-Fe-B alloy film, which is beneficial for the applications in spintronic devices design and optimization.

## 1. Introduction

The magnetization reversal due to spin-transfer-torque (STT) [1,2], spin-orbit-torque (SOT) [3], domain wall motion [4], and spin wave propagation [5] has been extensively studied with a view to designing spintronic devices that operate with less energy consumption and at faster speeds. Such magnetic phenomena are significantly affected by spin damping, which is characterized by the Gilbert damping constant *α* in the Landau–Lifshitz–Gilbert (LLG) equation [6]. Spintronic devices, for example, SOT magnetic random access memories (MRAMs), nano-oscillators, or magnonics favor a low damping parameter for small critical switching current and spin wave excitation [7,8,9]. Thus, it is desirable to engineer magnetic materials with a low damping and to fully understand the underlying mechanisms.

The damping studies of yttrium–iron–garnet (YIG) films [4,10,11] and Heusler alloys [12,13] were among the hottest topics in magnetism a few decades ago. In spite of such low damping from 10^−4^ to 10^−5^ [10,11], the complex oxides are hard to integrate with spintronic technologies. Meanwhile, high-quality YIG films and Heusler compounds typically require oxide substrates and high-temperature processing, which imposes a limitation on applications. Fortunately, conductive alloys provide an alternative solution. 

To date, the metallic ferromagnetic (FM) alloy Co-Fe-B has been widely used for magnetic layers in spintronic devices due to its perfect soft ferromagnetism, exhibiting controllable in-plane (IP) or out-of-plane (OOP) magnetic easy axis (MEA), high spin polarization, and considerable large tunneling magnetoresistance (TMR) in magnetic tunnel junctions (MTJs) [14,15,16,17]. It makes the study of low damping in Co-Fe-B alloy crucial to the design and optimization of spintronic devices. The damping of magnetic materials can always be modulated via composition [18], interface engineering [19], phase transition [20], external stimulus such as strain-mediated electric field [21], and thickness control. Regarding the numerous applications of Co-Fe-B alloy films in spintronic devices, thickness-dependent damping studies are attractive. It has been reported that stacks with Co-Fe-B/oxide interfaces exhibit a magnetic layer thickness-dependent damping rule. The aim of such a study is to produce high-efficiency spin currents through interface engineering [22,23,24] and to achieve low damping in memories with perpendicular magnetic anisotropy [25,26,27]. The complexity of this damping rule is attributed to interfaces of the Co-Fe-B film, as the Co-Fe-B/oxide interface can reduce the purity of the Co-Fe-B layer due to oxidation [28]. Alternatively, damping studies have been conducted in stacks without a Co-Fe-B/oxide interface [29,30,31]. However, in these stacks, the Co-Fe-B film is not fully protected by the seed layer and capping layer, thus exposing it to significant risk of oxidation from the atmosphere or the Si/SiO_2_ substrate. In the Co-Fe-B film sandwiched between non-oxide layers, damping studies are typically limited to a narrow thickness range of the magnetic layer, specifically no more than 10 nanometers (nm) [32,33]. In the meantime, the analysis of extrinsic Gilbert damping, such as radiative damping and eddy-current damping, usually relies on numerical calculations [34,35] in place of direct measurements. Consequently, the interplay between intrinsic and extrinsic dampings poses a major impediment to the accurate scrutiny of different damping mechanisms. In this regard, a universal thickness-dependent (several nm to tens of nm [36]) damping study and the development of an experimental method to fully understand the underlying mechanisms of low damping in stacks with Co-Fe-B film sandwiched by non-oxide layers is desirable. This is essential for advancing and refining the design of spintronic devices.

Here, we report on the thickness-dependent Gilbert damping and soft magnetism in metal/Co-Fe-B/metal sandwich structures. The capping layer and seed layer are the same in order to simplify the interfacial contribution analysis. In particular, in the context of multiple developments of spintronic devices, it is vital to explore the damping in the universal thickness range, ranging from 1 to 50 nm [36]. The chosen non-magnetic layer (NM) metallic layers for comparison are copper (Cu), molybdenum (Mo), tantalum (Ta), and platinum (Pt), which are defined as four series, taking the interfacial contribution from the Co-Fe-B/NM interface into account. By performing vibrating sample magnetometer (VSM) and ferromagnetic resonance (FMR) measurements, we demonstrate a non-monotonic thickness-dependent Gilbert damping rule in these sandwich structures. We have quantitatively disentangled the intrinsic and extrinsic Gilbert damping mechanisms and conducted magnetic anisotropy analysis to understand the strain effect in Co-Fe-B films. Our study demonstrates that the thickness-dependent damping measurement is an effective technique to explore the different damping mechanisms quantitatively. The minimum damping at a specific thickness and the intrinsic mechanism of low damping in the Co-Fe-B alloy film are helpful for spintronic devices design and optimization.

## 2. Experimental Details

**Figure 1:**
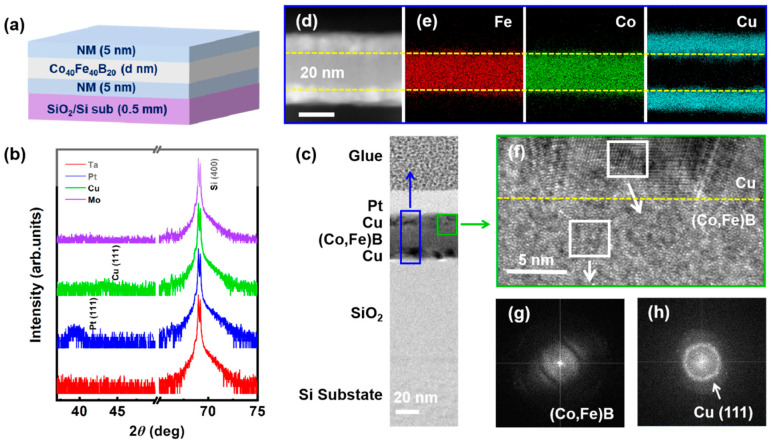
(**a**) Schematic of the sandwich structure deposited on the SiO_2_/Si substrate. (**b**) XRD pattern of Co-Fe-B films in each series. (**c**) Cross-section HRTEM image of Cu(5)/Co-Fe-B(20)/Cu(5)/SiO_2_/Si (100) substrate. The yellow dotted line shows the interfaces between the Cu layer and the Co-Fe-B layer. (**d**) A bright-field scanning TEM image taken in the region marked by blue rectangle in (**c**). (**e**) The corresponding EDS mapping for Fe, Co, and Cu element. (**f**) The high magnification HRTEM of the close-up region marked by the green rectangle in (**c**) and the FFT image (**g**,**h**) of the selected square regions.

The stacks of metal(5)/Co-Fe-B(*d*)/metal(5) for VSM and FMR measurements are deposited by a multisource high-vacuum magnetron sputtering system with a base vacuum of 1 × 10^−6^ Pa on Si/SiO_2_ substrate as shown in the schematic in Figure 1a (inside each parenthesis is the nominal thickness value). An IP magnetic field *H*_bias_ of about 50 Oe along one substrate’s edge is applied during the deposition to produce a foreseeable IP anisotropy. The Co-Fe-B denotes a nominal target composition of Co_40_Fe_40_B_20_. The surface roughness and the crystalline of the stacks are examined by NT-MDT NTEGRA scanning probe microscope (SPM) (Moscow, Russia) and Rigaku TTR-III High-power X-ray Diffractometer (XRD) (Tokyo, Japan), respectively. Energy-dispersive spectroscopy (EDS) mapping and fast Fourier transform (FFT) analysis in the high-resolution transmission electron microscopy (HRTEM) Tecnai G2 F30 S-Twin (Eindhoven, The Netherlands) are employed for studying the interface information and the stack’s nanostructure. Magnetization versus magnetic field (M-H) curves are measured by VSM (Model 3105, East Changing Technologies, Beijing, China). Spin dynamics properties are characterized in a home-built FMR spectrometer with a maximum magnetic field of 8 kOe and frequencies spanning from 4 to 26 GHz using an S-shape co-planar waveguide. All measurements are performed at room temperature. 

## 3. Results and Discussion

### 3.1. Structure Characterization

SPM and XRD are carried out to characterize the surface roughness and crystallization of the stacks. The roughness analysis in Supplementary Material Figure S1 indicates the flatness of the stack’s surface. Figure 1b shows no peaks from the Co-Fe-B alloy film, confirming the amorphous nature of the Co-Fe-B film. Surprisingly, the Pt (111) and Cu (111) peaks can be seen in the stacks, indicating the crystallization of the capping and seed layers. The absence of Ta and Mo peaks here is due to the X-ray detection limitation [37], combining the analysis of Ta(5)/Co-Fe-B(5)/Ta(5) in Supplementary Material Figure S2.

Cross-section EDS mapping and TEM analysis are performed to visually investigate the interface information and nanostructure of the representative stack Cu(5)/Co-Fe-B(20)/Cu(5). The HRTEM in Figure 1c displays the flat interface and ideal multi-layer structure. The colored rectangles mark the corresponding location of EDS (blue) and high-magnification HRTEM (green). Note that the actual thickness of each layer is consistent with the nominal value. The bright-field scanning TEM image (Figure 1d) and the corresponding EDS mapping (Figure 1e) for constituent elements reveal that Fe, Co, and Cu atoms are homogeneously distributed in each layer without any segregation at the interface. The interfaces between the Cu and Co-Fe-B are distinct as denoted by yellow dotted lines in Figure 1d–f. The high-magnification HRTEM presents the ordered Cu lattice and disordered Co-Fe-B atoms as shown in Figure 1f. The FFT focused on the Co-Fe-B layer and Cu layer is performed to confirm the nanostructure crystallization. A weak diffraction in Figure 1g and a plurality of diffraction rings in Figure 1h verify the amorphous Co-Fe-B film and polycrystalline Cu film in the stack, well matching what has been observed in the XRD measurement (Figure 1b).

A stack Ta(5)/Co-Fe-B(5)/Ta(5) is also characterized by EDS mapping and HRTEM for comparison as shown in Supplementary Material Figure S2. The FFT focused on the Co-Fe-B layer and Ta layer verifies the amorphous of Co-Fe-B film (Figure S2e) and the weak crystallization of Ta film (Figure S2f).

### 3.2. Soft Magnetism

**Figure 2:**
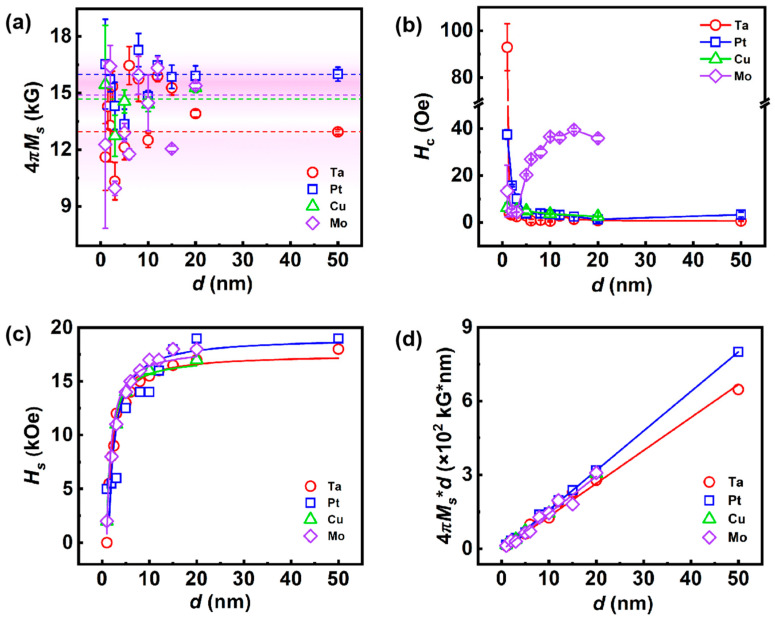
The collection of *M*_s_ (**a**) and *H*_c_ (**b**) for all stacks. The background color displays the statistical distribution of *M*_s_. The dotted lines denote the fitted values of each series from the bilayer model. (**c**) The thickness-dependent saturation field *H*_s_ (dots) and fitted by the demagnetization factor (lines) in the OOP direction. (**d**) The thickness-dependent sheet magnetization fitted with a bilayer model.

The M-H hysteresis loops for the different stacks are shown in Supplementary Material Figure S3. The saturation magnetization (*M*_s_) and coercivity (*H*_c_) of all stacks are collected in Figure 2a,b. The small saturation field (*H*_s_) of the IP M-H loop in Figure S3a–d indicates the MEA lies in the plane. The *M*_s_ of the stacks distribute in the range from 11 to 17 kG. The statistical distribution of *M*_s_ is denoted as the background color in Figure 2a, which is in agreement with the reported value (about 14–15 kG [38,39]). The *H*_c_ shows different rules in different series, which is decreasing versus the thickness increase for the Ta, Pt, and Cu series but increasing versus the thickness raise for the Mo series. The excellent soft magnetism is present in the stacks with thickness larger than 3 nm in Ta, Pt, and Cu series, in which *H*_c_ is less than 10 Oe. The OOP *H*_s_ is extracted from the M-H loops to evaluate the magnetic anisotropy. As shown in Figure 2c, the *H*_s_ increases with the rise of thickness and then turns to be gradually saturated when the thickness is larger than 20 nm. *H*_s_ can be well fitted by the thickness-dependent demagnetization factor equation [40]:

(1) $$\pi D_{z}=\left(p-\frac{1}{p}\right)ln\left(\frac{\sqrt{p^{2}+2}+1}{\sqrt{p^{2}+2}-1}\right)+\frac{2}{p}ln\left(\sqrt{2}+1\right)+pln\left(\frac{\sqrt{p^{2}+1}-1}{\sqrt{p^{2}+1}+1}\right)\;+2arctan\left(\frac{1}{p\sqrt{p^{2}+2}}\right)+\frac{2\left(1-p^{2}\right)}{3p}\sqrt{p^{2}+2}+\frac{2\left(1-p^{3}\right)}{3p}\;-\frac{2^{\frac{3}{2}}}{3p}+\frac{2}{3}\sqrt{p^{2}+1}\left(2p-\frac{1}{p}\right),$$

where $D_{z}$ is the demagnetizing factor in the OOP direction, and p is a geometry factor which is defined as the ratio of film thickness to width. Because the shape anisotropy energy in thin films is proportional to *D*_z_, *H*_s_ in our films can be well fitted by Equation (1), suggesting that *H*_s_ is affected by shape anisotropy.

The magnetic dead layer could originate from the intermixing of the metallic layer and Co-Fe-B layer at the interface and the oxidation of the FM layer [23]. In this regard, it is possible to fit the thickness-dependent *M*_s_ (Figure 2d) using a simple bilayer model to determine the dead layer’s thickness, i.e., *M*_s_*d = *M*_B_*(*d* − *d*_DL_) + *M*_DL_*d_DL_ [23,41], where *M*_B_ and *M*_DL_ represent the saturation magnetization of the bulk-like layer and dead layer, respectively. *d*_DL_ is the dead layer’s thickness. In that case, *M*_B_ = 16.1 ± 0.2 kG, 14.9 ± 0.3 kG, 15.0 ± 0.2 kG and 13.4 ± 0.2 kG for the Pt, Cu, Mo, and Ta series, respectively. The corresponding *M*_DL_ values are 0.7 ± 0.1 kG, 1.3 ± 0.3 kG, 0.6 ± 0.2 kG, and 1.1 ± 0.2 kG, respectively. The thickness of the dead layer, *d*_DL_ = 0.23 ± 0.02 nm, 0.40 ± 0.10 nm, 0.43 ± 0.11 nm, and 0.20 ± 0.05 nm, correspondingly. These results demonstrate that an ultra-thin magnetic dead layer with thickness less than 1 nm exists in our Co-Fe-B/NM interfaces [23,42]. In the following, these values of *M*_B_ will be employed as the effective *M*_s_ of each series. We will show that the results of spin-mixing conductance analysis in Section 3.3 match with the *d*_DL_ here, suggesting the magnetic dead layers originate from the intermixing in the Co-Fe-B/NM interfaces. 

### 3.3. Spin Dynamic Properties

**Figure 3:**
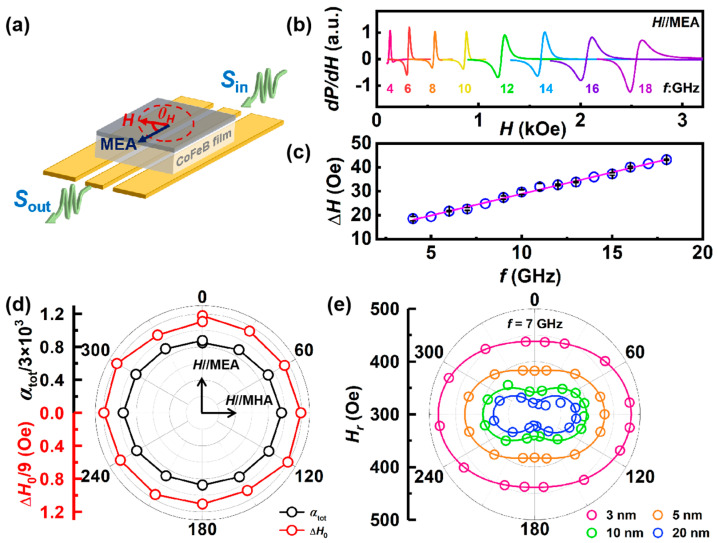
(**a**) Schematic of the FMR measurement for (**b**–**e**). The sample is laid face down on an S-shape co-planar waveguide. Magnetic fields are applied in the IP direction with the MEA (*θ*_H_). (**b**) Representative FMR spectra with frequency ranging from 4 to 18 GHz. (**c**) The linear frequency-dependent linewidth and (**d**) the *θ*_H_-dependent *α*_tot_ and Δ*H*_0_ measured at *f* = 7 GHz. (**e**) The *θ*_H_-dependent resonance field *H*_r_ of Cu/Co-Fe-B/Cu samples with different thickness measured at *f* = 7 GHz. The lines are fitted by Equation (7).

Next, broadband FMR measurement is carried out on all stacks to investigate the spin dynamic properties. The stack is faced down on an S-shape co-planar waveguide by which a microwave field with frequency (*f*) ranging from 4 to 26 GHz and IP magnetic field with variable direction are applied (Figure 3a). *θ*_H_ is the angle between the magnetic field direction and MEA, where the MEA direction is parallel to the *H*_bias_ during deposition. Figure 3b shows the typical FMR spectra of the Cu(5)/Co-Fe-B(3)/Cu(5) stack detected from 4 to 18 GHz. Each FMR spectrum can be accurately fitted using the Lorentz symmetric and antisymmetric functions [13]

(2) $$\frac{dP}{dH}=S\times \frac{4{∆H}^{2}(H-H_{r})}{{{[∆H}^{2}+4{\left(H-H_{r}\right)}^{2}]}^{2}}-N\times \frac{2∆H[{∆H}^{2}-4{\left(H-H_{r}\right)}^{2}]}{{{[∆H}^{2}+4{\left(H-H_{r}\right)}^{2}]}^{2}}+C,$$

where $\frac{dP}{dH}$ is the signal intensity, *H* is the applied magnetic field, *H*_r_ is the resonant field, *S* and *N* are the coefficients of Lorentzian symmetric and antisymmetric parts, Δ*H* is the FMR linewidth, and *C* is the offset. The extracted Δ*H* presents a linear relation with *f* as shown in Figure 3c, which can be fitted by the following equation [13,34]:

(3) $$∆H(f)=\frac{4\pi \alpha_{tot}}{\gamma }f+{∆H}_{0},$$

where *γ* = gµ_B_/ћ is the electron gyromagnetic ratio, *α*_tot_ is the total Gilbert damping constant and Δ*H*_0_ is the inhomogeneous linewidth broadening at 0 Hz. Since Δ*H* results from intrinsic and extrinsic contributions to damping, the *θ*_H_-dependent Δ*H* is measured to determine the direction of the applied magnetic field giving the minimal Δ*H* value, where the extrinsic contributions to the linewidth are minimal. *θ*_H_ = 0 means the MEA direction. As shown in Figure 3d, the isotropy of Δ*H*_0_ and *α*_tot_ indicates there is no specific direction of Δ*H* and *α*_tot_, which is different from the anisotropy of *H*_r_ (Figure 3e). The frequency-dependent Δ*H* along MEA is then employed as shown in Supplementary Material Figure S4 to determine the *α*_tot_ of all stacks. 

**Figure 4:**
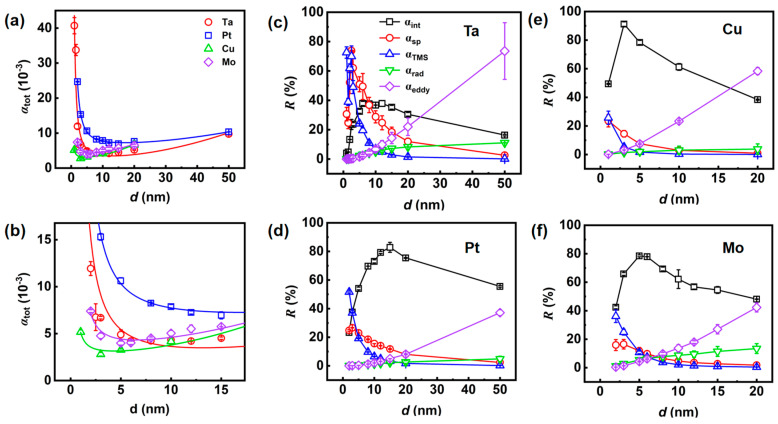
(**a**) The thickness-dependent *α*_tot_ in all stacks. The lines are fitted by Equation (4). (**b**) The enlargement of the specific region of (**a**). (**c**–**f**) The relative contributions *R* of each mechanism in Ta, Pt, Cu, and Mo series, respectively.

Figure 4a reveals a non-monotonic thickness-dependent *α*_tot_ rule in all stacks. Figure 4b is the enlargement of the thickness region from 0 to 15 nm. Actually, *α*_tot_ consists of intrinsic and extrinsic damping contributions [34], wherein the latter is usually caused by interfacial contributions such as spin pumping, two-magnon scattering (TMS), radiative damping, and eddy current. All extrinsic damping mechanisms are thickness-dependent. The intrinsic damping (*α*_int_) reflects an inherent characteristic of the magnetic material that is not affected by the thickness of the film. Interfacial contributions including spin memory loss [43], interfacial isotropic scattering [44], and spin pumping [13,21,22,24] are phenomenologically inverse in film thickness with the coefficient *β*_sp_ = *α*_sp_*d, where *α*_sp_ is the damping of interfacial contribution and *d* is the FM film thickness. The TMS arises when a uniform FMR mode is destroyed and degenerate magnons of different wave vectors are created [45]. The momentum non-conservation is accounted for by considering a pseudo-momentum derived from the internal field inhomogeneities or secondary scattering. Recently, TMS has been found to be the dominant contribution to damping in heavy metal/FM heterostructures, and this finding provides further justification for the *d*^−2^ dependence of the TMS term (*α*_TMS_) [46]. At last, as we measure the damping using an FMR with a conductive co-planar waveguide (Figure 3a), spin precession in the FM layer induces AC currents both in the FM layer and the co-planar waveguide. The dissipation of these AC currents within the stacks and the flow of energy into the co-planar waveguide both give rise and contribute to damping. Historically, the damping caused by eddy currents in the FM layer *α*_eddy_ is called eddy-current damping, while the induced damping in the waveguide is called radiative damping *α*_rad_. *α*_eddy_ is quadratically proportional to the film thickness (*α*_eddy_ = *β*_eddy_*d^2^), while *α*_rad_ scales linearly with the film thickness (*α*_rad_ = *β*_rad_*d) [34,35]. Therefore, the *α*_tot_ value is given by the sum of the five damping mechanisms as [47]

(4) $$\alpha_{tot}=\alpha_{int}+\alpha_{sp}+\alpha_{TMS}+\alpha_{rad}+\alpha_{eddy}\;=\alpha_{int}+\frac{\beta_{sp}}{d}+\frac{\beta_{TMS}}{d^{2}}+\beta_{rad}*d+\beta_{eddy}*d^{2},$$

where *β*_sp_, *β*_TMS_, *β*_rad_, and *β*_eddy_ are the corresponding coefficients of each mechanism. 

All coefficients are listed in Table 1. Concerning the $\alpha_{rad}=\frac{\gamma \mu_{0}^{2}M_{s}ld}{16ZW}$, where *µ*_0_ is the vacuum permeability, *l* is the length of the stack, *Z* = 50 Ω is the impedance and *W* = 100 µm is the width of the waveguide, our fitted value of *α*_rad_ is consistent with the one calculated by the formula [34,35]. It has been suggested that *α*_rad_ is anisotropic and only works with perpendicular FMR geometry [34,35]. A disentanglement without *α*_rad_ is also carried out as shown in Supplementary Material Figure S5. The comparison of the coefficients between Table 1 and Table S1 indicates little difference. The relative contributions *R* of each mechanism are plotted in Figure 4c–f for different series. It can be seen that the *α*_rad_ contributes no more than 10% even in the thick films. By contrast, the *α*_eddy_ varies enormously. The *α*_eddy_ can be negligible in the thickness less than 5 nm but becomes extremely large in thick films. The *α*_tot_ enhancement in thick films mainly comes from the contribution of *α*_eddy_, as observed by Li et al. [48]. Since *α*_rad_ and *α*_eddy_ represent energy consumption in the FMR facility, we define a critical thickness *d*_cri_ as the sum of these two contributions exceeds the remaining three, allowing us to compare internal and external energy consumption. As shown in Table 1, the *d*_cri_ is larger than 20 nm in the Pt and Ta series but becomes smaller in the Mo and Cu series. This rule indicates that the heavy metal plays a role in reducing the external energy consumption. In addition, the minimum damping *α*_min_ (Table 1) at the specific thickness *d*_min_ of each series provides a reference for low-damping spintronic device design.

More intrinsic information can be obtained when we consider the difference of *α*_int_, *α*_sp_ and *α*_TMS_ in different series. Regarding the TMS mechanism in the previous introduction [46], the good linear relationship between (*β*_TMS_)^1/2^ and perpendicular magnetic anisotropy field (*H*_⟂_) as analyzed in Supplementary Material Figure S6 indicate the TMS mechanism originates from the interfacial perpendicular magnetic anisotropy in our stacks. The perpendicular magnetic anisotropy density of NM/FM interface *K*_s_ = 1.18 erg/cm^2^ in Pt(5)/Co-Fe-B(3)/Pt(5) is slightly larger than the previous report [46] because of the double Co-Fe-B/Pt interfaces here. Note that the stack’s MEA lies in the plane despite there being a large *H*_⟂_ in Co-Fe-B/Pt interface, which means other IP magnetic anisotropies exist. Apart from TMS, the principal impact on the interface comes from the spin pumping effect, in which an external stimulation incites a precession of magnetization within the FM layer. This precession of magnetization leads to a buildup of spins resting at the NM/FM interface. A neighboring NM layer, which functions as an ideal spin sink, collects these spins using spin-flip scattering, resulting in a significant increase in the Gilbert damping parameter of FM. The spin-pumping effect has received significant attention for producing a high-efficiency spin current [13,22,24]. According to spin pumping theory, the movement of spin across the NM/FM interface is directly influenced by the spin-mixing conductance. This conductance has two types, namely, (a) *g*_↑↓_, which excludes the effect of spin angular momentum back-flow, and (b) *g*_eff_, which includes the back-flow impact. The spin channel conductive property attribute at the NM/FM interface is represented by the spin-mixing conductance, which can be described by the ballistic spin transport model [22,24]

(5) $$g_{eff}=g_{↑↓}(1-e^{-\frac{2t}{\lambda_{eff}}})=\frac{4\pi M_{s}}{g\mu_{B}}d\times \alpha_{sp},$$

(6) $$\alpha_{sp}=\frac{g\mu_{B}}{4\pi M_{s}d}\times g_{↑↓}(1-e^{-\frac{2t}{\lambda_{eff}}}),$$

where *t* is the thickness of the NM metallic layer, and *λ*_eff_ is the effective spin diffusion length in the Co-Fe-B/NM interface. Referring to the small value of *λ*_eff_ [13,49] and *t* = 5 nm in our stacks, the contribution of spin angular momentum back-flow is negligible. Taking the value of *M*_B_ extracted from the bilayer model fitting and *g* = 2.15 [49], the analysis of *β*_sp_ leads to *g*_↑↓_ = 8.36 ± 0.11 nm^−2^, 9.84 ± 0.13 nm^−2^, 0.92 ± 0.02 nm^−2^, and 1.77 ± 0.03 nm^−2^ for Ta, Pt, Cu, and Mo series, respectively. Our results are comparable with the references [22,24]. The Co-Fe-B/Cu and Co-Fe-B/Mo interfaces display lower values of *g*_↑↓_ compared to the Co-Fe-B/Pt and Co-Fe-B/Ta interfaces. The cause of this trend can be traced back to the intermixing that occurs at the Co-Fe-B interfaces, which creates a wider interface region. This wider region may neutralize the sudden potential variation at the interfaces, making it less likely for conducting electrons to scatter and, consequently, resulting in a decrease in interface spin losses. The high efficiency of spin pumping in Co-Fe-B/Pt and Co-Fe-B/Ta interfaces indicates the strong Co-Fe-B interfacial spin-flip scattering, which is attributed to the large spin-orbit coupling and spin-flip scattering parameter in heavy metal [50]. We emphasize here that the stronger intermixing in Co-Fe-B/Cu and Co-Fe-B/Mo interfaces is consistent with the *d*_DL_, confirming the intermixing mechanisms in dead layer’s formation as discussed in Section 3.2.

The thickness-dependent spin pumping contribution *R*_sp_ is non-monotonic in Ta and Pt series but shows a decreasing rule with the thickness rise in Cu and Mo series (red curves in Figure 4c–f). Actually, *R*_sp_ is a non-monotonic curve with Co-Fe-B thickness *d*, which is written as *R*_sp_ *= α*_sp_/*α*_tot_ = *β*_sp_/(d*α_tot_). The thickness-turning point *d*_tr_ can be found by the differentiation of *R*_sp_, producing an equation 3*d_tr_^4^*β_eddy_ *+* 2*d_tr_^3^*β_rad_ *+ d*_tr_^2^*α_int_ *= β*_TMS_. There must be an appropriate thickness satisfying the equation for each series. For Ta and Pt series, the comparatively large *β*_TMS_ makes the *d*_tr_ locate in the thickness range of 1 to 5 nm, which can be observed in our measurement. However, the small *β*_TMS_ in the Cu and Mo series decreases the *d*_tr_ to less than 1 nm, leading to the monotonic *R*_sp_ with thickness larger than 1 nm.

### 3.4. Magnetic Anisotropy

**Figure 5:**
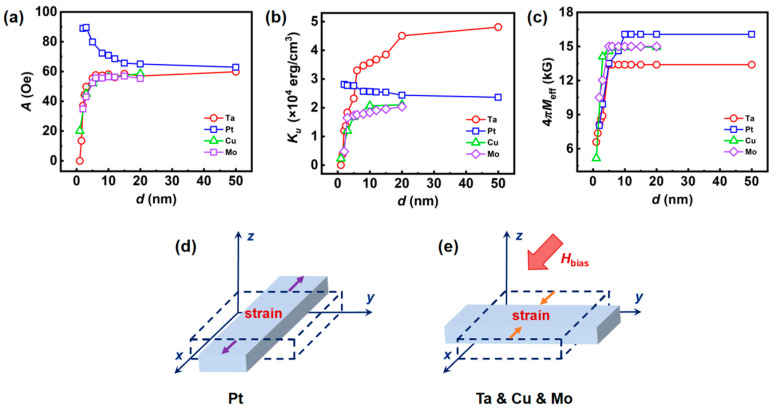
(**a**) The thickness-dependent anisotropy intensity *A*. The thickness-dependent IP uniaxial magnetic anisotropy coefficient *K*_u_ (**b**) and effective magnetization *M*_eff_ (**c**). (**d**,**e**) The schematic of the Co-Fe-B film under tensile stress and compressive stress. The dash lines represent the film’s original volume.

Finally, we briefly discuss the magnetic anisotropy in our stacks measured by FMR. It has been shown that all stacks exhibit IP uniaxial magnetic anisotropy with MEA along the direction of *H*_bias_ during deposition (Figure 3e and Supplementary Material Figure S7). The anisotropy of *H*_r_ can be fitted using [51]

(7) $$H_{r}=A\times {sin}^{2}(\theta_{H})+B,$$

where *A* and *B* are anisotropy intensity and offset, respectively. As shown in Figure 5a, the anisotropy intensities increase with Co-Fe-B thickness in Ta, Cu, and Mo series rather than the decreasing trend in Pt series. The IP anisotropy variation with Co-Fe-B thickness could be verified by IP uniaxial magnetic anisotropy coefficient (*K*_u_), which is extracted from *f*-dependent *H*_r_ fitting in the Kittel equation [13,49]

(8) $${\left(\frac{\omega }{\gamma }\right)}^{2}=(H_{r}+\frac{2K_{u}}{M_{s}})\times (H_{r}+4\pi M_{eff}+\frac{2K_{u}}{M_{s}}),$$

where $4\pi M_{eff}=4\pi M_{s}-{µ}_{0}H_{⟂}$ is the effective magnetization, *H*_⟂_ is the perpendicular magnetic anisotropy field as discussed in Section 3.3, and *ω* = 2πf.

The thickness-dependent *K*_u_ and *M*_eff_ are shown in Figure 5b,c, respectively. Since both *K*_u_ and *A* represent the magnitudes of magnetic anisotropy, Figure 5a,b confirm that the magnetic anisotropy increases with Co-Fe-B thickness in the Ta, Cu, and Mo series, but it decreases in the Pt series. Generally, magnetic anisotropy can originate from magnetocrystalline anisotropy, induced anisotropy, shape anisotropy, interfacial anisotropy, and strain effect [51]. Magnetocrystalline anisotropy can be ruled out due to the amorphous nature of the Co-Fe-B film as shown in XRD and TEM. Induced anisotropy is caused by *H*_bias_ during deposition, which is the same in all stacks. Shape anisotropy is usually a function of the geometry factor, as described in Equation (1). Since the geometry of all stacks is indistinguishable, the shape anisotropy in the stack is the same. Interfacial anisotropy usually is helpful for perpendicular magnetic anisotropy as predicted by Néel [52]. It is dominant in ultra-thin films with thickness less than 1 nm and suppressed by shape anisotropy in thicker films. Although there is *H*_⟂_ in our stacks, the competition between *H*_⟂_ and the demagnetization field makes the MEA lie in the plane. Thus, IP uniaxial magnetic anisotropy in our stacks is the sum of induced anisotropy, shape anisotropy, interfacial anisotropy, and strain effect. Combining the analysis on the induced anisotropy, shape anisotropy and interfacial anisotropy in our stacks, the strain effect could be the only reason for the opposite trend of *K_u_* and *A* as seen in Figure 5a,b.

Strain effect is universal in stack samples, which could originate from the residual stress of the substrate, lattice mismatch, crystallization of the NM layer and sample clamping [53,54,55,56]. The strain effect can affect the thickness of the magnetic film up to several hundred nm [57] and decreases with thickness increasing [54]. Since the magnetic anisotropy in our stacks is IP uniaxial magnetic anisotropy with the MEA along the direction of *H*_bias_, the stress direction should be parallel to the *H*_bias_. The contribution of the strain effect in the Pt series (Ta, Cu, Mo series) should assist (suppress) the magnetic anisotropy, suggesting opposite strain effects as the schematics shown in Figure 5d,e. Now, we conclude the stain is not from the substrate and the deposition, since we use the identical substrate and deposition process for all samples. The strain is probably related to NM layers because they are opposite in the Pt series to the Ta, Cu, and Mo series.

## 4. Conclusions

In summary, we have investigated the thickness-dependent Gilbert damping and soft magnetism of the Co-Fe-B film in the metal/Co-Fe-B/metal sandwich structure. The structure characterization confirms the amorphous nature of the Co-Fe-B film and the crystallization of the metallic NM film. The flat interfaces from EDS mapping demonstrate the ideal sandwich structure, avoiding the risk of Co-Fe-B oxidation. Soft magnetism study shows the *M*_s_, *H*_s_ and dead layer of each series. Performing co-planar waveguide FMR measurements reveals a non-monotonic thickness-dependent Gilbert damping rule in this structure. Significantly, *α*_int_, *α*_sp_, *α*_TMS_, *α*_rad_, and *α*_eddy_ are quantitatively disentangled. The TMS mechanism originates from the interfacial perpendicular magnetic anisotropy at film thicknesses less than *d*_cri_, while *α*_eddy_ dominates the contribution of *α*_tot_ in the films at film thicknesses greater than *d*_cri_. In addition, the high-efficiency of spin pumping in Co-Fe-B/Pt and Co-Fe-B/Ta interfaces is related to the large spin-orbit coupling and spin-flip scattering parameters in heavy metal. Based on the magnetic anisotropy analyses, we conclude that the IP uniaxial magnetic anisotropy of the stacked layers is the sum of the induced anisotropy, interfacial anisotropy, shape anisotropy, and strain effect, and that there are opposite strain effects in the Pt series to the Ta, Cu, and Mo series. Our results suggest that thickness-dependent damping measurements are effective for quantitatively exploring various damping mechanisms. The intrinsic mechanism of low damping in Co-Fe-B alloy films and the minimum value at specific thicknesses is beneficial for improved and processed spintronic devices for applications.

## Figures and Tables

**Table 1:** Thickness-dependent *α*_tot_ fitting to disentangle the coefficients of each damping mechanism. NM stands for the NM/Co-Fe-B/NM sandwich structure.

NM	*α*_int_ (10^−3^)	*β*_sp_ = *α*_sp_ × *d* (10^−2^ nm)	*β*_TMS_ = *α*_TMS_ × *d*^2^ (10^−2^ nm^2^)	*β*_rad_ = *α*_rad_/*d* (10^−5^ nm^−1^)	*β*_eddy_ = *α*_eddy_/*d*^2^ (10^−6^ nm^−2^)	*d*_min_/*α*_min_ (nm/10^−3^)	*d*_cri_ (nm)
Ta	1.59	1.25	2.95	2.16	2.87	12.4/3.49	24.0
Pt	5.75	1.22	5.10	1.00	1.54	16.1/7.26	>50.0
Cu	2.55	0.12	0.13	1.26	9.72	4.54/3.14	15.8
Mo	3.13	0.24	1.07	4.36	6.89	6.29/4.32	18.9

## Data Availability

Data are contained within the article and supplementary materials.

## Supplementary Materials

The following supporting information can be downloaded at https://www.mdpi.com/article/10.3390/nano14070596/s1, Figure S1: Topography image of representative stacks Ta/Co-Fe-B/Ta and Pt/Co-Fe-B/Pt; Figure S2: EDS mapping and TEM of stack Ta(5)/Co-Fe-B(5)/Ta(5) for comparison; Figure S3: IP and OOP M-H hysteresis loops of the stacks; Figure S4: The *f*-dependent linewidth Δ*H* of the stacks; Figure S5: A disentanglement of intrinsic and extrinsic damping contributions without radiative damping α_rad_, the coefficients of each damping mechanism are listed in Table S1; Figure S6: Analysis of relationship between TMS mechanism and perpendicular magnetic anisotropy field *H*⟂; Figure S7: IP *H*_r_ anisotropy for Co-Fe-B film in Ta, Pt, and Mo series.
