# Supplementary material for: Thickness-Dependent Gilbert Damping and Soft Magnetism in Metal/Co-Fe-B/Metal Sandwich Structure
Source: Nanomaterials (Basel). 2024 Mar 28;14(7):596. doi: 10.3390/nano14070596 (PMC11013670; doi:10.3390/nano14070596)
Supplement: Supplementary file 1 [file nanomaterials-14-00596-s001.zip › nanomaterials-2923401-supplementary.pdf]

Supplementary Material:

Thickness-dependent Gilbert damping and soft magnetism in metal/Co-Fe-B/metal sandwich structure

Yimo Fan[1], Jiawei Wang[1,2,*], Aitian Chen[3], Kai Yu[2], Mingmin Zhu[2], Yunxin Han[4], Sen Zhang[4], Xianqing Lin[1], Haomiao Zhou[2], Xixiang Zhang[3], and Qiang Lin[1,*]

[1] College of Science, Zhejiang University of Technology, Hangzhou, 310023, China

[2] Key Laboratory of Electromagnetic Wave Information Technology and Metrology of Zhejiang Province, College of Information Engineering, China Jiliang University, Hangzhou, 310018, China

[3] Physical Science and Engineering Division, King Abdullah University of Science and Technology, Thuwal, 23955-6900, Saudi Arabia

[4] College of Science, National University of Defense Technology, Changsha, 410073, China

S1. Topography information of the stacks.

Scanning probe microscope (SPM) is performed to characterize the roughness at the stack's surfaces in the representative samples. Except distinct dust spots, no specific pattern could be observed in these topography images. The Root-Mean-Square (RMS) roughness is less than 2 nm, indicating the flatness of the surface.

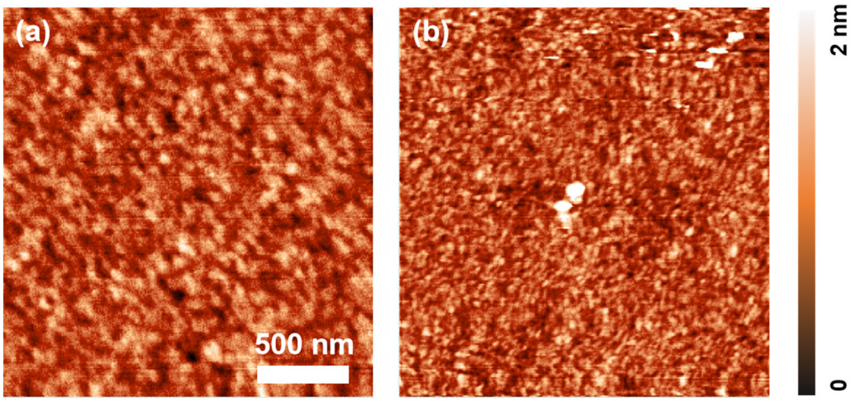

Figure S1. Topography image of stack Ta/Co-Fe-B/Ta (a) and Pt/Co-Fe-B/Pt (b) with Co-Fe-B film's thickness 50 nm.

S2. Cross-section energy-dispersive spectroscopy (EDS) mapping and transmission electron microscopy (TEM) of stack Ta(5)/Co-Fe-B(5)/Ta(5).

Cross-section EDS mapping and TEM are also performed in a stack Ta(5)/Co-Fe-B(5)/Ta(5). The flat interface and ideal multi-layer structure are apparent in HRTEM figure S2(a). The thickness of each layer is consistent with the nominal value. The rectangles here mark the corresponding location of EDS (left) and high magnification HRTEM (right). The bright-field scanning TEM image (figure S2(b)) and the corresponding EDS mapping (figure S2(c)) for constituent element reveal that Fe, Co, and Ta atoms are homogeneously distributed in each layer. The flat up and down interfaces are

denoted by cyan solid lines in figures S2(b)-S2(d). The fast Fourier transform (FFT) focused on the Co-Fe-B layer and Ta layer are performed in the corresponding square in the high magnification HRTEM, respectively (figure S2(d)). A weak diffraction in figure S2(e) and diffraction spot in figure S2(f) verify the amorphous Co-Fe-B film and polycrystalline Ta film in the stack.

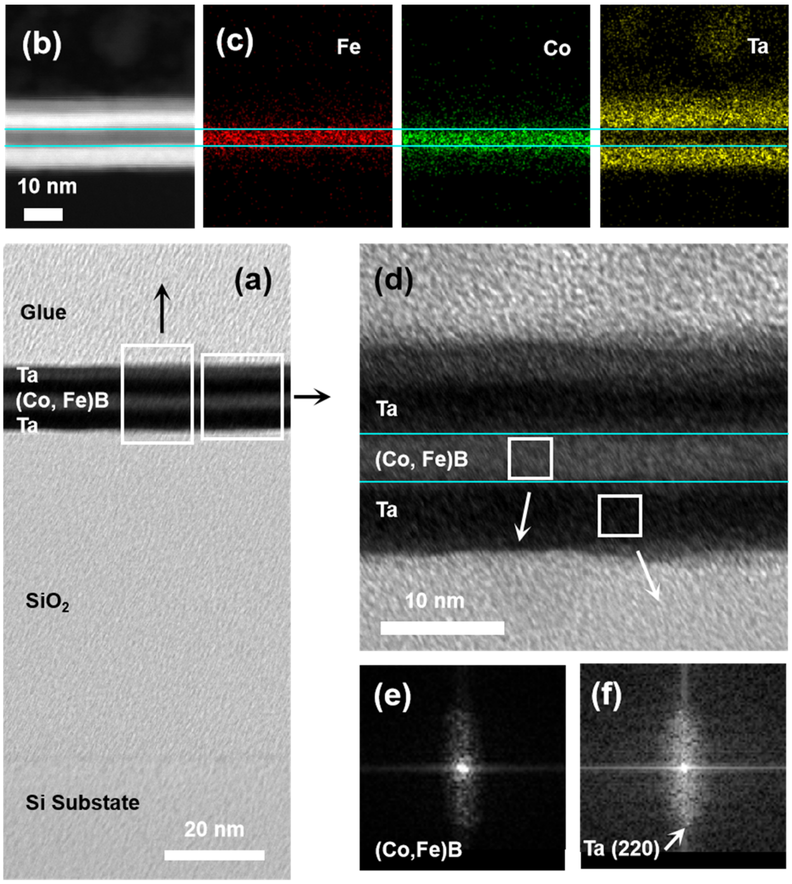

Figure S2. (a) Cross-section HRTEM image of Ta(5)/Co-Fe-B(5)/Ta(5)/SiO_2/Si (100) substrate. (b) A bright-field STEM image taken in the region marked by rectangle in (a). (c) The corresponding energy-dispersive x-ray spectroscopy mappings for Fe, Co, and Ta element. (d) The high magnification HRTEM of the local region marked by rectangle in (a) and FFT image(e-f) of the selected square regions in Co-Fe-B layer and Ta layer.

S3. The in-plane (IP) and out-of-plane (OOP) M-H hysteresis loops for Co-Fe-B film with Ta, Pt, Cu, and Mo non-magnetic layer (NM).

The IP and OOP M-H hysteresis loops are measured by vibrating sample magnetometer (VSM) with scanning field between +2 kOe and -2 kOe along IP and OOP direction, respectively. The loops are manifested as scanning field between ±1 kOe (figures S3 (a)-(d)) and between ±0.2 kOe (figures S3 (e)-(h)) for clearness.

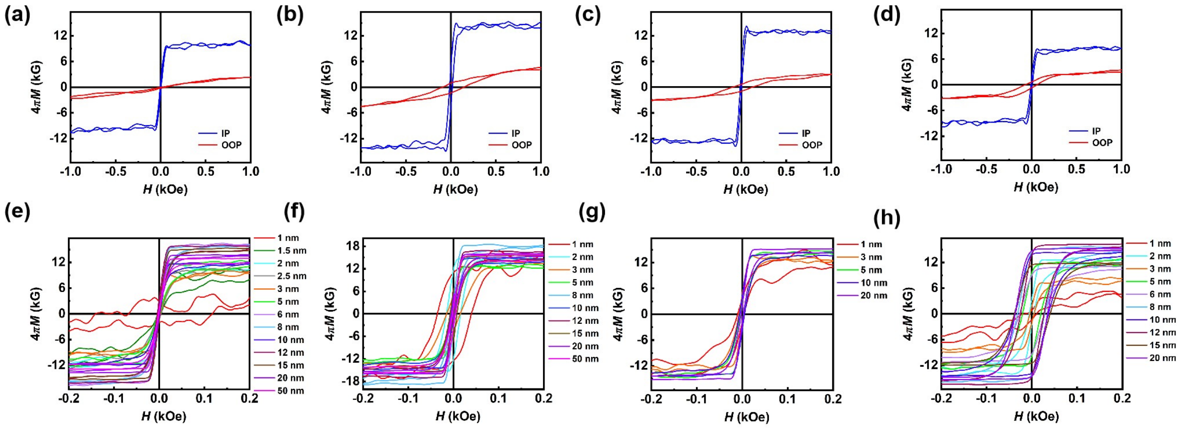

Figure S3. (a-d) M-H curves taken in IP and OOP configuration of a representative 3-nm-thick Co-Fe-B film in Ta, Pt, Cu, and Mo series, respectively. (e-h) IP M-H curves of different thickness Co-Fe-B films in Ta, Pt, Cu, and Mo series, respectively.

S4. The frequency-dependent linewidth (ΔH) along the magnetization easy axis (MEA) direction.

To determine the total Gilbert damping α_{tot} of all stacks, the ΔH extracted from Eq. (2) versus frequency is plotted as figures S4(a)-(d). The α_{tot} is obtained by fitting with Eq. (3). The inset in figure S4(a) is the result for 1-nm-thick Co-Fe-B film in Ta series.

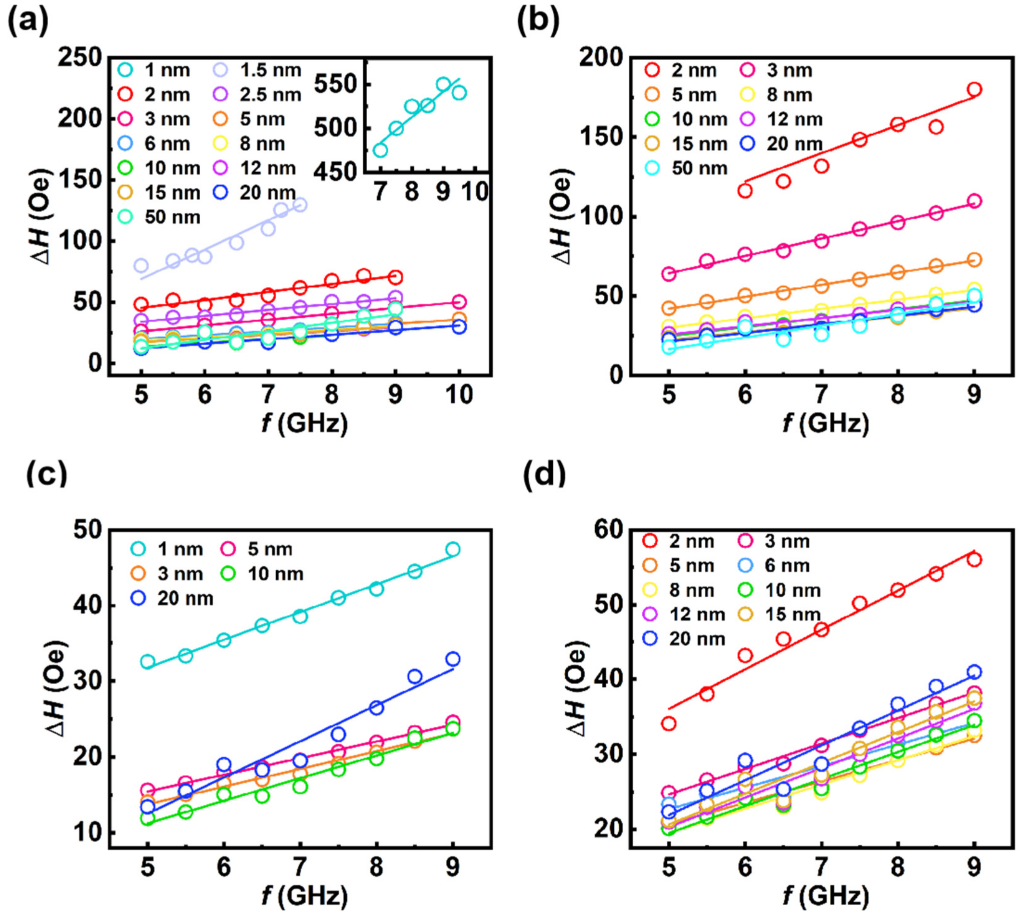

Figure S4. (a-d) The frequency-dependent ΔH of different thickness Co-Fe-B films in Ta, Pt, Cu, and Mo series, respectively. The inset in (a) is the data of 1-nm-thick Co-Fe-B film in Ta series, which shows obviously large α_{tot}.

S5. A disentanglement of intrinsic and extrinsic damping contributions without radiative damping (α_{rad}).

It has been suggested that α_{rad} is anisotropic and only work with perpendicular FMR geometry [1, 2]. Regarding the IP FMR geometry in our measurement, a disentanglement without α_{rad} is also carried out here for comparison.

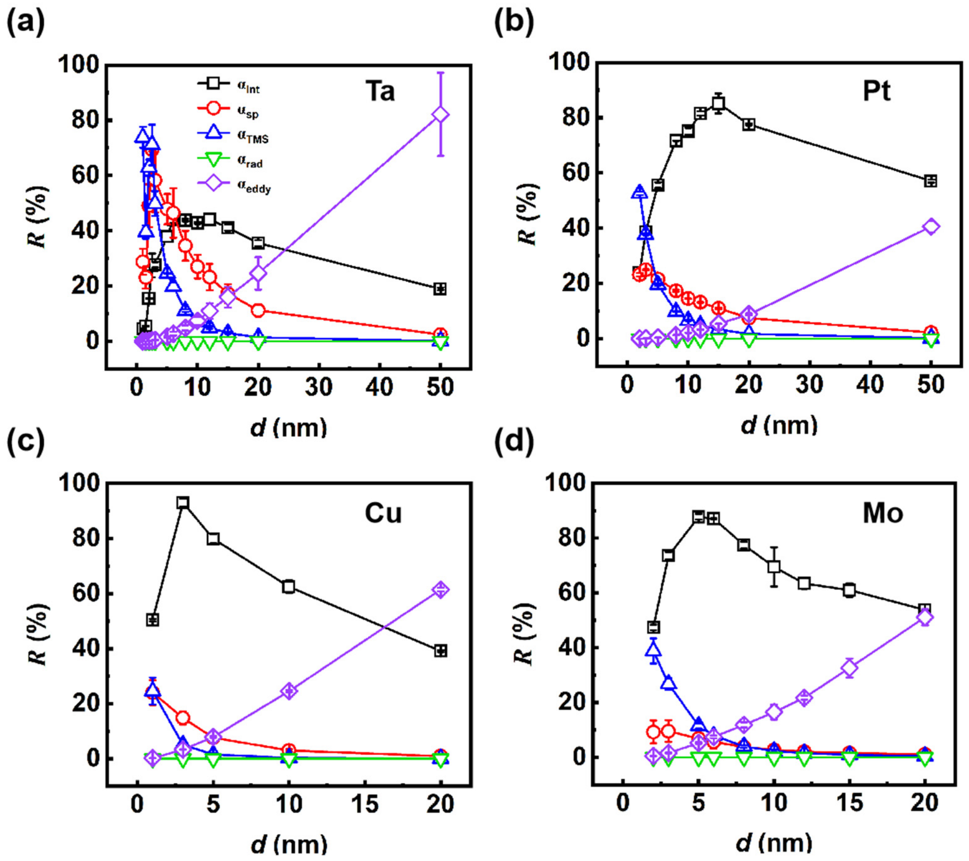

Figure S5. (a-d) The relative contributions R of each mechanism in Ta, Pt, Cu, and Mo series, respectively. Here, the α_{rad} is not included in disentanglement.

NM	α_{int} (10^{-3})	$\beta_{sp}=\alpha_{sp}*d$ (10^{-2} nm)	$\beta_{TMS}=\alpha_{TMS}*d^2$ (10^{-2} nm^2)	$\beta_{rad}=\alpha_{rad}/d$ (10^{-5} nm^{-1})	$\beta_{eddy}=\alpha_{eddy}/d^2$ (10^{-6} nm^{-2})	d_{min}/α_{min} (nm/10^{-3})	d_{cri} (nm)
**Ta**	1.86	1.17	3.00	0	3.21	13.1/3.47	27.0
**Pt**	5.91	1.15	5.20	0	1.68	16.4/7.25	> 50.0
**Cu**	2.60	0.12	0.13	0	10.2	4.10/3.15	16.2
**Mo**	3.50	0.13	1.15	0	8.32	6.50/4.33	20.8

Table S1. The coefficients of each damping mechanism in a disentanglement without α_{rad}. The α_{int}, α_{sp}, α_{TMS}, and α_{eddy} are the corresponding coefficient of intrinsic, spin pumping, two-magnon scattering (TMS) and eddy-current mechanism.NM stands for NM/Co-Fe-B/NM sandwich structure.

S6. Analysis of relationship between TMS mechanism and perpendicular magnetic anisotropy field ($H_{\perp}$).

As previously discussed [3], to the first approximation, α_{TMS} is a coefficient that depends on both $(\frac{2K_s}{M_s * d})^2$ and the density of magnetic defects at the ferromagnetic (FM) surfaces, where K_s denotes perpendicular magnetic anisotropy density of NM/FM interface, M_s is the saturation magnetization and d is the thickness of Co-Fe-B film. Since perpendicular magnetic anisotropy field can be obtained according to Eq. (8) and there are similar structure roughness in different stacks (as shown in figure S1), we plot the $(\beta_{TMS})^{1/2}$ versus $H_{\perp}=2K_s/(M_s*d)$ here. The stacks with 3-nm-thick Co-Fe-B film are chosen to ensure the TMS contributes significantly in each stack. The good linear relationship between $(\beta_{TMS})^{1/2}$ and $H_{\perp}$ confirms the parabolic function between α_{TMS} and $H_{\perp}$.

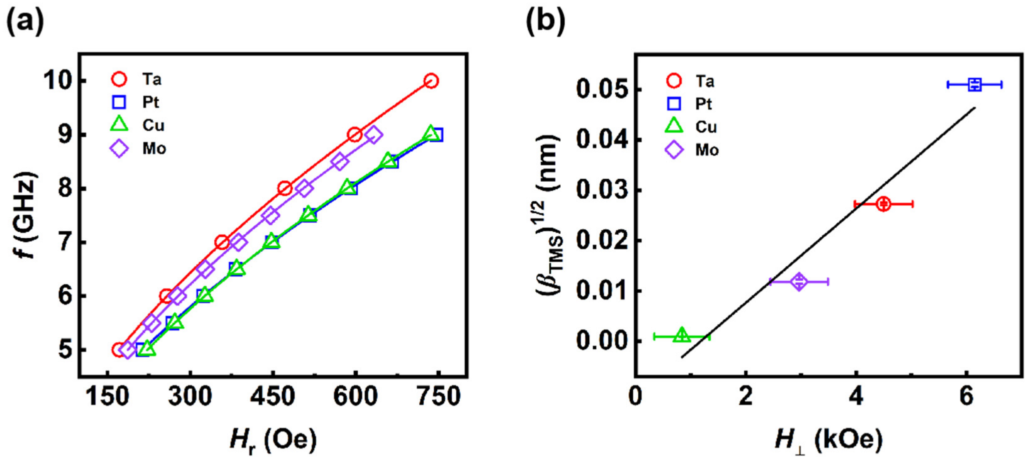

Figure S6. (a) f-dependent H_r fitting by Eq. (8) in stacks with 3-nm-thick Co-Fe-B film to obtain the $H_{\perp}$. (b) The linear relationship between $(\beta_{TMS})^{1/2}$ and $H_{\perp}$.

S7. IP H_r anisotropy for Co-Fe-B film in Ta, Pt, and Mo series.

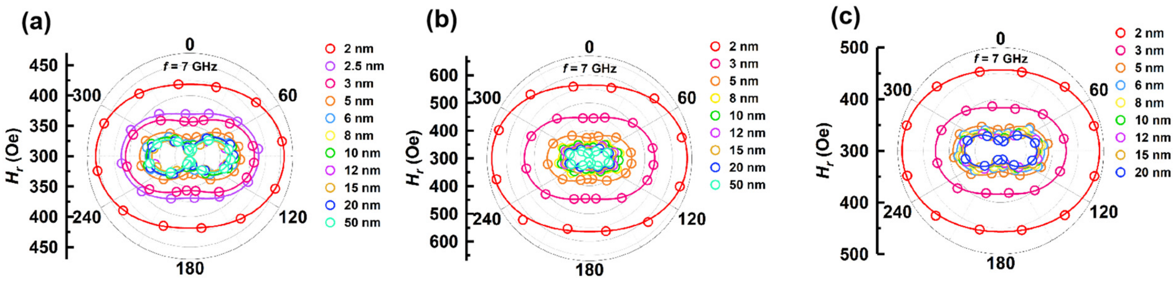

Figure S7. (a-c) The θ_H-dependent resonance field H_r of different thickness Co-Fe-B films in Ta, Pt, and Mo series, respectively, which are measured at f = 7 GHz. The lines are fitted by Eq. (7).

References

[S1] Schoen M A W; Thonig D; Schneider M L; Silva T J; Nembach H T; Eriksson O; Karis O; and Shaw J M. Ultra-low magnetic damping of a metallic ferromagnet. *Nature Phys*. **12**, 839 (2016).

[S2] Schoen M A W; Shaw J M; Nembach H T; Weiler M; and Silva T J. Radiative damping in waveguide-based ferromagnetic resonance measured via analysis of perpendicular standing spin waves in sputtered permalloy films. *Phys. Rev. B* **92**, 184417 (2015).

[S3] Zhu L; Ralph D C; and Buhrman R A. Effective Spin-Mixing Conductance of Heavy-Metal-Ferromagnet Interfaces. *Phys. Rev. Lett.* **123**, 057203 (2019).
